# Accuracy of Classifying Lung Carcinoma Using Immunohistochemical Markers on Limited Biopsy Material: A Two-Center Study

**DOI:** 10.7759/cureus.32956

**Published:** 2022-12-26

**Authors:** Amber Hassan, Shadi Alahmadi, Omer Waqas, Humaira Waseem, Amer Shafie Abdelrahman, Majid Almansouri, Nasser Mulla, Yousef Katib, Salwa I Bakhsh, Mohammed Basheikh, Samy A Abusikkien, Mohamed Matoog Karami, Marwan Al-Hajeili, Samah S Elbasateeny

**Affiliations:** 1 Translational Neuroscience Lab, CEINGE-Biotecnologie Avanzate, Naples, ITA; 2 European School of Molecular Medicine, University of Milan, Milan, ITA; 3 Department of Anatomic Pathology, Faculty of Medicine, King Abdulaziz University, Jeddah, SAU; 4 Department of Pathology, Ittefaq Hospital (Trust), Lahore, PAK; 5 Department of Research/Biostatistics, Fatima Jinnah Medical University, Lahore, PAK; 6 Department of Pathology, Faculty of Medicine in Rabigh, King Abdelaziz University, Rabigh, SAU; 7 Department of Clinical Biochemistry, Faculty of Medicine, King Abdulaziz University, Jeddah, SAU; 8 Department of Internal Medicine, Faculty of Medicine, Taibah University, Medina, SAU; 9 Department of Radiation Oncology/Internal Medicine, Faculty of Medicine, Taibah University, Medina, SAU; 10 Department of Pathology, Faculty of Medicine, King Abdulaziz University, Jeddah, SAU; 11 Department of Medicine, Faculty of Medicine, King Abdulaziz University, Jeddah, SAU; 12 Department of Anatomy, Faculty of Medicine in Rabigh, King Abdulaziz University, Rabigh, SAU; 13 Department of Anatomy, Faculty of Medicine, Ain Shams University, Cairo, EGY; 14 Department of Physiology, Faculty of Medicine, King Abdulaziz University, Jeddah, SAU; 15 Department of Oncology, Faculty of Medicine, King Abdulaziz University, Jeddah, SAU; 16 Department of Pathology, Faculty of Medicine in Rabigh, King Abdulaziz University, Rabigh, SAU; 17 Department of Pathology, Zagazig University, Zagazig, EGY

**Keywords:** squamous cell carcinoma, adenocarcinoma, small cell carcinoma, sensitivity, specificity, immunohistochemistry, lc, trucut biopsy

## Abstract

Introduction

Accurate classification of lung cancer into primary and metastatic carcinomas is critical for treatment approaches. Immunohistochemistry (IHC) has always been pivotal in unveiling the diverse cell differentiation lineages present in lung cancer by using specific biomarkers such as TTF1 and p63/p40, which closely reflect the relationship between genotype and phenotype..

Methods

A retrospective cross-sectional study was conducted to evaluate 57 Tru-Cut biopsies over two years, from 2020-2022. Tumour morphology was evaluated, and IHC for TTF-1, Napsin A, CK-7, P-63, P-40, and CD-56 was performed in two steps.

Results

Of the lung cancer cases, 58.5% were adenocarcinoma (ADC), 24.5% were squamous cell carcinoma (SCC), 9.4% were small cell carcinoma, and 7.5% were poorly differentiated carcinoma. TTF1 stain had sensitivity and specificity of 78.9% and 50% in 33 cases of ADC, respectively, while CK7 and Napsin A had 100% sensitivity. P63 stain had 77% sensitivity and 50% specificity in 15 cases of SCC, while P-40 had 100% sensitivity. The CD56 stain was 100% sensitive in five cases of small cell carcinoma.

Conclusion

IHC staining on small lung biopsies allows accurate sub-classification of poorly differentiated lung cancers; however, there is still significant variability. Surgical resection specimens can be further classified due to architectural features that biopsies lack. Morphological findings would be beneficial in the development of an algorithm for sub-classifying lung carcinoma using a variety of markers.

## Introduction

Globally, lung cancer (LC) is reported as the leading cause of mortalities. It is known that 85% of LC cases belong to non-small cell lung cancer (NSCLC) [1]. According to the WHO guidelines, the most common histological subtypes of NSCLC are adenocarcinoma (ADC) (50-70%), squamous cell carcinoma (SqCC) (20-30%), small cell carcinoma (SCC), and poorly differentiated carcinoma (PDC) [2]. The importance of histological subtyping at diagnosis in LC treatment has recently been highlighted, not only in determining the best therapeutic strategy but also in avoiding serious side effects [3]. We have seen an evolution in diagnostic techniques for LC over the years, but despite all efforts, only 15% of stage I tumours and 60% of stage II tumours have the prognosis rate. Imaging techniques helped in diagnosing and staging LC [4]. However, advances in molecular biology and immunohistochemistry (IHC) have significantly improved our understanding of lung tumours [5]. Recently, epidermal growth factor receptor (EGFR) and tyrosine kinase inhibitors (TKI) such as gefitinib and erlotinib [6], are reported to be effective only in tumours with EGFR kinase domain mutations that are most common in ADC [7].

The morphologic assessment of routinely stained histological sections has always been the primary basis for LC classification, but this approach may be difficult or even impossible in cytological preparations or small biopsies. Accurate histopathological diagnosis on a biopsy material is difficult due to the inadequate availability of the tissue. Tru-Cut biopsy (TCB) and fine needle aspiration (FNA) achieve comparable rates of definitive and accurate LC diagnosis and subtyping of NSCLC. Optimal results are attained when the two modalities are considered jointly. In soft tissue mass diagnosis, TCB is more accurate than FNA on all accounts, establishing the exact diagnosis, and guiding appropriate treatment [8]. Because of the scarcity of tumour tissue in FNA specimens, distinguishing ADC from SqCC can be difficult. In terms of histological information, however, TCB outperforms FNA. Because surgical resection is not an option for patients with advanced-stage NSCLC at the time of diagnosis, the majority (70%) of LC are identified and evaluated using only adequate biopsy specimens [9]. Despite ongoing research efforts, no ideal IHC diagnostic method has yet emerged, although its importance in this context has been consistently emphasized. However, several IHC markers, including tumour protein 63 (P63) and cytokeratins 5 and 6, commonly expressed in SCC, as well as thyroid transcription factor 1 (TTF-1) and cytokeratin 7 in ADC, have been proposed to differentiate between major NSCLC subtypes [10]. The most accurate and sensitive squamous division marker has been P-40 [11]. Several studies have evaluated the accuracy of IHC staining using tissue microarrays [12]. There hasn't been much discussion in the literature about how IHC on the TCB determines the correct classification in these cancers [13].

We retrospectively assessed the efficacy of TTF-1, CK7, P63, P40, and Napsin A, IHC markers in differentiating and sub-classifying ADC, NSCLC, and SCC on TCBs to determine the accuracy of the tumour markers. TCB is important in diagnosing and staging primary and metastatic LC. Accurate ADC and SqCC sub-classification is critical for targeted therapy, but it is difficult in small TCB specimens [14]. Although Napsin A has been proposed as a potential new marker of lung ADC [15], few researchers have found it to be less sensitive than TTF-1, and its specificity is also lacking, staining up to 26% of SCC [16]. TTF-1 is now regarded as the best single stain for lung ADC, with the added benefit of being a pneumocystis jiroveci pneumonia marker [17]. Colorectal cancer (CRC) is responsible for a significant proportion of lung metastasis, which may resemble primary lung ADC [18] Furthermore, tissue management strategies have recently been emphasized to determine a precise diagnosis as well as to allow for future molecular testing. CK5/6, p63, and p40 are equally effective in differentiating SqCC from carcinoid tumours.TTF-1, Napsin A, CK-7, and CD-56 IHC markers were used to distinguish and subclassify ADC, SqCC, and SCC [19]. Several studies have evaluated the accuracy of IHC staining for classifying lung carcinoma using tissue microarrays derived from resection specimens [20].

The literature has paid little attention to the question of how frequently IHC markers on TCB in lung tumours lead to the correct classification. To differentiate between primary and metastatic lung adenocarcinoma, we intended to create a panel of IHC markers that would allow more accurate histopathological subtyping of NSCLC. To achieve this, we examined the chosen markers on several LC biopsies and correlated the outcomes to the diagnosis established on the biopsies and, when available, the resection specimen.

## Materials and methods

Case selection 

This is a retrospective cross-sectional study approved by the Institutional Review Board, The Diagnostic Center, Lahore, Pakistan (approval number: IRB/680-I/2020). The clinicopathological data was compiled from the laboratory-based software (LIMS) annual report and were characteristics, using gender and age groups. The two-centre study involved all patients diagnosed with LC at the Department of Histopathology and Radiology, Diagnostic Centre, Lahore, Pakistan, and the Department of Pathology, Ittefaq Hospital, Lahore, Pakistan from June 1, 2020, to June 30, 2022. Patients' medical and personal histories including age, disease duration, tumour site/size, disease progression, and staging/grading, were recorded. We followed the American Joint Committee on Cancer Staging and End Results Reporting guidelines for data collection. The 57 TCB were identified. The search yielded 33 cases of ADC of the lung, 13 cases of SqCC, seven cases of SCC, and four cases of PDC. The clinical information and available slides were reviewed. All research cases were documented with any relevant clinical data that was available.

Immunohistochemistry (IHC) staining assay 

As part of the routine clinical evaluation of these cases, IHC was performed on paraffin-embedded tissue segments. The tissue sections of five microns thickness were placed onto charged slides, dewaxed, rehydrated in pH 7.5 buffer, and processed for standard IHC staining. Antibodies of TTF1 (M3575, 1:100), Napsin-A (EPR6252, 1:4000), CK-7 (GA619, 1:200), P63 (GA662,1:200), P40 (GA784,1:800), CD56 (M7304, 1:100) were obtained from Agilent Dako (Santa Clara, California, United States) and used at the manufacturer's recommended concentrations. Microscopy was performed using an Olympus (Model U-DO3; Olympus Corporation, Shinjuku City, Tokyo, Japan).

Grading of IHC stains

Review and consideration were given to the immunostaining features as well as the staining patterns' distribution and intensity. If more than 5% of tumour cells with the proper staining pattern were discovered, the case was deemed positive; otherwise, it was considered negative. Regarding particular staining patterns, Napsin A was found in coarse granular cytoplasmic staining. TTF-1, CD56, and P63 nuclear staining were considered positive. Cytoplasmic staining for CK7 was considered positive. Each assay had appropriate positive and negative controls. Positive staining was not interpreted as entrapped normal lung bronchial epithelium or pulmonary macrophages. TTF-1, Napsin A, CK-7, P-63, P-40, and CD-56 immunostaining results were associated with the tumour's histological diagnosis. Validity measures were calculated, including sensitivity, specificity, positive predictive value (PPV), negative predictive value (NPV), and accuracy.

Statistical analysis 

The data was entered and analyzed by using IBM SPSS Statistics for Windows, Version 23.0 (Released 2015; IBM Corp., Armonk, New York, United States). All the quantitative variables were reported in frequency and percentage. Specificity, sensitivity, NPV, PPV, and diagnostic accuracy were calculated while using IHC as a gold standard.

## Results

Patient characteristics

After excluding patients with missing information from the study, 57 patients were considered. Out of the total, 66% were males and 34% were females. The ages of the patients ranged from 40-87 years with a majority of the cases in the sixth and seventh decades. The peak incidence was seen in the sixth decade of life. The maximum number of reported cases was of NSCC, accounting for 50 cases (87.7%) while seven cases (12.2%) were of SCC. Among the 50 cases of NSCC, 33 cases (66%) were of ADC, 13 cases (26%) cases were of SqCC, and four cases (8%) were of PDC (Table 1).

**Table 1:** Clinicopathological characteristics NSCC: non-small cell carcinoma; SCC: small cell carcinoma; ADC: adenocarcinoma; SqCC: squamous cell carcinoma; PD-SCLCL poorly differentiated small cell lung carcinoma

Variables	n	%
Patient’s demographics		
Males	38	66%
Females	19	34%
Age range	40-87 years	
Grade		
I (well diff)	35	61.4%
II (mod)	18	31.5%
III (poor)	4	7.0%
Pathology T Staging		
PT1	5	8.7%
PT2	30	52.6%
PT3	16	28.0%
PT4	6	10.5%
Histological Type of Tumor		
NSCC	50	87.7%
SCC	7	12.2%
Classification of NSCC		
ADC	33	66%
SqCC	13	26%
PD-SCLC	4	8%

Expression of CK7, p40, p63 in NSCLC

**Figure 1:**
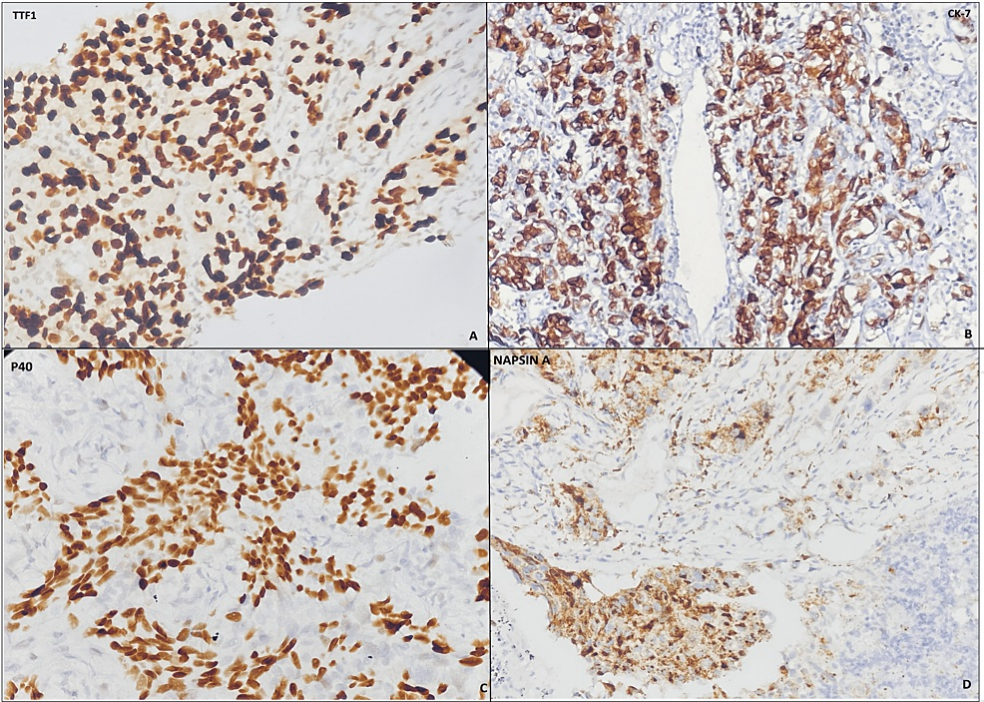
Photograph of IHC panel: (A) IHC of TTF1 shows the nuclear positivity in tumour cells; (b) CK-7 shows cytoplasmic staining in tumour cells; (C) P40 shows nuclear and cytoplasmic staining; (D) Napsin-A shows granular cytoplasmic and nuclear staining in tumour cells IHC: immunohistochemistry

The results of IHC staining on TCB specimens for each histologic subtype of carcinoma, and the sensitivity, specificity, PPV, and NPV of the IHC stains for ADC and SqCC were evaluated respectively. P63 IHC stain was positive in 10 out of 13 cases (77%) of histopathological diagnosed SqCC. It was positive in eight out of 16 cases (50%) of adenocarcinoma and negative in all four of four cases of SCC (Table 2). In our study, the sensitivity of the P63 IHC stain for SqCC was 77%, specificity was 50%, PPV was 55.5%, NPV was 72.3%, and accuracy was 62% (Table 3). The P40 IHC stain was performed on six cases of SqCC and showed positive staining in all six out of six cases of SqCC making the sensitivity of P40 100% (Tables 2, 4). CK7 was positive in 20 out of 20 cases of ADC making the sensitivity 100% (Table 2)(Figure 1).

**Table 2:** Immunohistochemical findings on histopathological diagnosis of TCB The total number of TCB cases for each histological type and the total number of positive cases for the antibodies studied. TCB: Tru-cut biopsy; ADC: adenocarcinoma; SqCC: squamous cell carcinoma; SCC: small cell carcinoma; IHC: immunohistochemistry

Histopathological diagnosis of TCB						
	Biopsy IHC number positive/ total stained					
	TTF1	P63	P40	CK7	Napsin A	CD 56
ADC	28/33	8/16	-	20/20	15/15	2/2
SqCC	3/6	10/13	6/6	-	-	-
SCC	1/7	-	-	-	-	7/7

**Table 3:** Sensitivity, specificity, positive predictive value, and negative predictive value of TTF1 and Napsin A in Tru-cut lung biopsies

	TTF1	Napsin A
Sensitivity	84%	100%
Specificity	50%	-
Positive predictive value	90%	100%
Negative predictive value	37.5%	-

**Table 4:** Sensitivity, specificity, PPV, and NPV of IHC stains P63 and P40 in TCB lung PPV: positive predictive value; NPV: negative predictive value; TCB: Tru-cut biopsy; IHC: immunohistochemistry

	P63	P40
Sensitivity	77%	100%
Specificity	50%	-
Positive predictive value	55.5%	100%
Negative predictive value	72.7%	-

Expression of TTF1, Napsin A, and CD56 in NSCLC

TTF1 IHC staining was positive in 28 of the 33 ADC cases. It was positive in three of six SqCC cases, one of four PDC, and one of seven SCC cases (Table 2). In our study, the sensitivity of TTF1 IHC stain for ADC was 84%, specificity was 50%, PPV was 90%, NPV was 37.5%, and accuracy was 78.38%, represented to be a specific marker for ADC (Table 3). The Napsin A IHC stain was performed on 15 out of 33 cases of ADC (due to limited biopsy material in the remaining 18 cases). It showed positive staining in all 15 of the 15 cases (100%) of ADC making the sensitivity of Napsin A 100% (Tables 2, 3). CD56 IHC stain was positive in seven out of seven cases of SCC and two out of two cases of adenocarcinoma. The sensitivity of the CD56 IHC stain was 100% for SCC, and the accuracy was 71.4% (Table 2) (Figure 1).

## Discussion

A revolution in the field of lung cancer is underway, largely driven by therapeutic advances, with significant implications for pathologic diagnosis and tissue management. Because of their advanced stage, 70% of lung cancers cannot be resected. The majority of lung cancer patients continue to be diagnosed using small biopsy specimens [21]. This study set out to determine the effectiveness of IHC stains that are usually practised in diagnosing LC in the Pakistan region. In general, ADC is diagnosed with morphological findings of acinar/tubular structures or mucin production as well as IHC-positive markers for TTF-1 and/or Napsin-A and SCC is defined by keratinization or intercellular bridges with associated IHC markers such as P-40, P-63, and CK-5/CK-6, with P-40 being the most sensitive and specific.

Recently, the classification of LC, particularly the non-small cell variant, was discovered to be very important in targeted therapy, so an accurate subtype is critical, especially in small biopsy specimens [22]. The International Association for the Study of Lung Cancer (IASLC) has recommended the use of immune stains as a diagnostic aid, particularly in tumours that do not meet established morphologic criteria by hematoxylin and eosin (H&E) stain [23]. The established morphological criteria for ADC are glandular differentiation or mucin, whereas intercellular bridges and/or keratinization are the criteria for SCC [24]. The performance of IHC is recommended if morphological criteria are absent. On the other hand, biopsy specimens frequently have classification issues because of inadequate sampling or the existence of a very little quantity of tumours that might not exhibit differentiated characteristics. The current study findings show that certain IHC markers have high sensitivity and specificity for specific subtypes of lung tumours, which can aid in subtyping tumours in limited biopsy material. Although many of these stains have previously been reported to be useful, most studies have focused on resection specimens [25], tissue microarrays [26], or a combination of specimen types rather than specifically on biopsy specimens [27].

Since most lung tumours at the time of diagnosis and biopsy are sometimes the only tissue available, we focused on biopsy specimens. The most common type of cancer in our study was NSCLC, which accounted for 50 cases (87.7%), while SCC accounted for seven cases (12.2%). NSCC was also identified as the most common lung tumour in studies [28]. There were 33 cases (66%) of ADC, 13 cases (26%) of SqCC, and four cases (8%) of PDC among the 50 cases of NSCLC. ADC was also identified as the most common NSCLC [28,29].

In the current work, TTF-1, Napsin A, p40, p63, and CD56 showed high sensitivity and specificity for specific lung tumours. The sensitivity of the P-63 IHC stain for SqCC in our study was 77%, specificity was 50%, PPV was 55.5%, NPV was 72.3% and accuracy was 62%. Literature also shows a high sensitivity of p63 IHC stain for diagnosing SqCC of the lung [30]. The low specificity of the P-63 IHC stain (50%) was due to its positive staining in eight cases of ADC. A similar P-63 IHC stain for diagnosing SqCC was also seen in the reported literature. P-40 IHC stain showed a high sensitivity for diagnosing SqCC, i.e. 100%. CD56 and CK7 IHC stains showed 100% sensitivity for diagnosing SCC and ADC respectively. In our study, we found that the Napsin A IHC stain showed a sensitivity of 100% for diagnosing ADC as compared to the TTF1 IHC stain, which showed a sensitivity of 84%. International studies also show a similarly high sensitivity of Napsin A in diagnosing ADC as compared to TTF1 [30]. We can combine TTF-1, Napsin A, and p40 markers in one panel to differentiate NSCLC as reported in recent work [30]. Literature supports the importance of the CK5/6 marker but It is not surprising that CK5/6 is expressed in some pulmonary ADC because CK5/6 is present in normal basal cells of the respiratory epithelium.

The study conducted by Rossi reported the panel of four antibodies including TTF1, CK7, CD56, and 34bE12 to classify the pulmonary LCC in the bronchial biopsy. The results indicate that TTF-1, CK7, and CD56 were all negative for SqCC, while 34bE12 was positive for ADC. TTF-1 and CD56 were negative in LCC and CK7 and 34bE12 were positive in large-cell neuroendocrine carcinomas [30]. The use of p63 in this context may be important because it is a nuclear marker, whereas 34bE12 is a cytoplasmic marker that can give false-negative results depending on the degree of preservation of the tissue used.

In this study, we chose a TCB representation of the tumour's histological pattern as sufficient to test an IHC panel for routine purposes. The well-known morphological heterogeneity of NSCLCs raises the question of whether IHC staining on small biopsies is representative of the resected tumour. The IHC staining using a combination of LC markers enables accurate sub-classification of weakly differentiated NSCLCs on small lung specimens in the great majority of cases. However, the limitation of our study was we did not evaluate whole tissue because of inadequate tissue samples, and to avoid tissue depletion, few cases were selected; the limitations of IHC in small biopsies of morphologically unclassifiable tumours are greater than are commonly acknowledged because there is a definite "miss" rate when using IHC to predict resection tumour type, and subsequent surgical resections change the diagnosis in a significant minority of cases, almost entirely based on morphology rather than new IHC staining patterns.

The most important aspect of this work in the future would be to use these immunohistochemical tests on transbronchial biopsy specimens or some other type of small specimen with the same results obtained on resection specimens and then use this information to guide treatment. In the near future, evaluating LC for gene mutations, gene amplification, tumour-related angiogenesis, DNA repair gene expression levels, and genomic or proteomic profiles will be an exciting challenge for pathologists.

## Conclusions

In our study, the diagnostic accuracy of IHC markers for sub-classifying lung carcinomas varied. Morphological findings can help in the development of a procedure for a sub-classification of LC by combining various markers. IHC staining on small lung biopsies allows accurate sub-classification of LCs; however, there is still significant variability. TTF-1, Napsin A, p63, and CK7 can accurately subtype NSCLCs on small lung biopsies. ADCs should be defined as TTF-1-positive tumours with focal p63 staining. Surgical resection specimens can be further classified due to architectural features that biopsies lack. Morphological findings would be beneficial in the development of an algorithm for sub-classifying LC using a variety of markers and WHO criteria for classifying PDCs of the lung should be considered.
